# Enhanced Triacylglycerol Content and Gene Expression for Triacylglycerol Metabolism, Acyl-Ceramide Synthesis, and Corneocyte Lipid Formation in the Epidermis of Borage Oil Fed Guinea Pigs

**DOI:** 10.3390/nu11112818

**Published:** 2019-11-18

**Authors:** Ju-Young Lee, Kwang-Hyeon Liu, Yunhi Cho, Kun-Pyo Kim

**Affiliations:** 1 Department of Medical Nutrition, Graduate School of East-West Medical Science, Kyung Hee University, Gyeonggi-do 17104, Korea; bell1924@naver.com; 2 BK21 Plus KNU Multi-Omics Based Creative Drug Research Team, College of Pharmacy and Research Institute of Pharmaceutical Sciences, Kyungpook National University, Daegu 41566, Korea; dstlkh@knu.ac.kr

**Keywords:** borage oil, triacylglycerol metabolism, acyl-ceramide, corneocyte lipid envelope, epidermis

## Abstract

Triacylglycerol (TAG) metabolism is related to the acyl-ceramide (Cer) synthesis and corneocyte lipid envelope (CLE) formation involved in maintaining the epidermal barrier. Prompted by the recovery of a disrupted epidermal barrier with dietary borage oil (BO: 40.9% linoleic acid (LNA) and 24.0% γ-linolenic acid (GLA)) in essential fatty acid (EFA) deficiency, lipidomic and transcriptome analyses and subsequent quantitative RT-PCR were performed to determine the effects of borage oil (BO) on TAG content and species, and the gene expression related to overall lipid metabolism. Dietary BO for 2 weeks in EFA-deficient guinea pigs increased the total TAG content, including the TAG species esterified LNA, GLA, and their C20 metabolized fatty acids. Moreover, the expression levels of genes in the monoacylglycerol and glycerol-3-phosphate pathways, two major pathways of TAG synthesis, increased, along with those of TAG lipase, acyl-Cer synthesis, and CLE formation. Dietary BO enhanced TAG content, the gene expression of TAG metabolism, acyl-Cer synthesis, and CLE formation.

## 1. Introduction

Triacylglycerol (TAG) is one of the most abundant lipids in the epidermis, and TAG synthesis and hydrolysis are reported to be related to acyl-ceramide (Cer) synthesis and corneocyte lipid envelope (CLE) formation [1,2,3]. The systemic lack of diacylglycerol O-acyltransferase 2 (*Dgat2*), the gene for TAG synthesis, leads to an impaired permeability barrier in the epidermis [4]. In addition, mutation of *CGI-58*, the gene that facilitates TAG hydrolysis, has been reported to induce severe ichthyosis along with impaired CLE formation [3]. In parallel, the epidermal levels of linoleic acid (LNA; 18:2n-6) esterified acyl-Cer, a key lipid involved in CLE formation, were decreased in cases of both impaired permeability barriers and ichthyosis [2,3], suggesting that LNA, which is hydrolyzed from TAG, could be required for acyl-Cer synthesis and CLE formation in the differentiated epidermis.

In *Dgat2*-deficient mice, the level of LNA esterified to TAG is reduced, and a skin phenotype is exhibited similar to that seen in essential fatty acid (EFA) deficiency, which is characterized by a scaly skin condition and increased trans-epidermal water loss (TEWL) [4]. Interestingly, levels of TAG are reduced in the epidermis of EFA-deficient mice [5]. In addition, γ-linolenic acid (GLA; 18:3*n*-6), another ω-6 EFA, has been reported to exert greater efficacy than LNA in recovering EFA deficiency in the epidermis [6]. Furthermore, our recent studies demonstrated that GLA is also esterified to acyl-Cer in the epidermis [7]. Therefore, we hypothesized that there is a relationship among LNA or GLA esterified TAG, CLE formation, and epidermal barrier homeostasis. To test this hypothesis, we performed lipidomic and transcriptome analyses to determine the effect of dietary BO containing high concentrations of GLA and LNA on TAG content and TAG metabolism related gene expression levels.

## 2. Materials and Methods

### 2.1. Animals

Male albino Hartley guinea pigs at 3 weeks of age were purchased from the Samtako Laboratory (Osan, Republic of Korea) and housed as described previously [8]. After 1 week adaptation period, EFA deficiency was induced by a diet containing 40 g/kg hydrogenated coconut oil (HCO) (400950; Dyets, Bethlehem, PA, USA) supplemented with 20 g/kg triolein (T7752) (Sigma-Aldrich, St Louis, MO, USA) for 8 weeks. Subsequently, EFA deficient guinea pigs were fed a diet containing 60 g/kg BO (Midlands Seed, Ashburton, New Zealand) (group HCO + BO; *n* = 5) or continued on the HCO diet (group HCO: EFA deficient control; *n* = 5) for 2 weeks. Animal care and handling was approved by the Animal Care and Use Review Committee of Kyung Hee University, Yongin, Republic of Korea (KHUAGC-17-020).

### 2.2. Lipidomic Analysis

The epidermal lipid was extracted using Folch solution (chloroform/methanol, 2:1, *V*/*V*) and high-performance thin-layer chromatography (HPTLC) was used for analysis of the total epidermal TAG content as described previously [9]. Lipidomic analysis was performed using direct infusion electrospray mass spectrometry (ES-MS) as described previously [10]. Specifically, epidermal lipid samples with chloroform/methanol (1:9, *V*/*V*) containing 7.5 mM ammonium acetate were infused into a Thermo LTQ XL ion trap mass spectrometer (Thermo Fisher Scientific, West Palm Beach, FL, USA) through a nanoelectrospray infusion system (TriVersa NanoMate, Advion Biosciences, Ithaca, NY, USA). The spectral data were recorded using the Thermo Xcalibur software (version 2.1, Thermo Fisher Scientific) and loaded into the Genedata Expressionist MSX module (Genedata AG, Basel, Switzerland) for data processing. After subtracting background noise, spectral data below an intensity of 300 were removed, and data alignment was performed using nonlinear transformation to map the original *m*/*z* data onto the common universal data. After spectrum peak detection, isotopic clustering of individual detected peaks was conducted using spectrum isotope clustering activity. After normalizing the spectral data, each compound was identified using commercially available standards or the Lipidomics Gateway of the LIPID MAPS (http://www.lipidmaps.org).

### 2.3. Transcriptome Analysis

The total RNA was extracted using TRIzol reagent (Gibco, New York, NY, USA) from the epidermis of a randomly selected subpopulation of 3 guinea pigs from each group, HCO and HCO + BO. The RNA was amplified, labelled with Cyanine 3 using the Quick AMP Labeling Kit (Agilent Technologies, Santa Clara, CA, USA), and purified. The labeled complementary RNA was hybridized to an Agilent 4 × 180K Custom Gene Expression Microarray. After washing, microarrays were scanned, and data were extracted with Feature Extraction 9.1 Software (Agilent Technologies, Santa Clara, CA, USA). Subsequently, the gene expression data were quantile-normalized and log2-transformed.

To identify the alteration of epidermal transcriptomes between groups HCO and HCO + BO, we extracted gene ontology (GO) terms from the genes (*n* = 1243) whose expression in group HCO + BO was 1.5-fold higher than that in group HCO, using a web-based gene set analysis toolkit (www.webgestalt.org) [11]. Next, to identify the alteration of TAG metabolism, we screened genes associated with the TAG biosynthetic process with a 2-fold change (FC) threshold. The expression levels of the selected genes were confirmed by quantitative reverse transcription polymerase chain reaction (qRT-PCR) using specific primers (Table 1) as described previously [8]. Lastly, we screened genes associated with TAG hydrolysis, acyl-Cer synthesis, and CLE formation to gain insight into the meaning of the altered TAG species.

### 2.4. General Statistical Analysis

All data are expressed as means ± standard deviation, and differences between groups HCO and HCO+BO were tested by unpaired Student’s *t*-test. *p*-values < 0.05 were considered significant.

## 3. Results and Discussion

### 3.1. Altered TAG Content and Species

**Figure 1:**
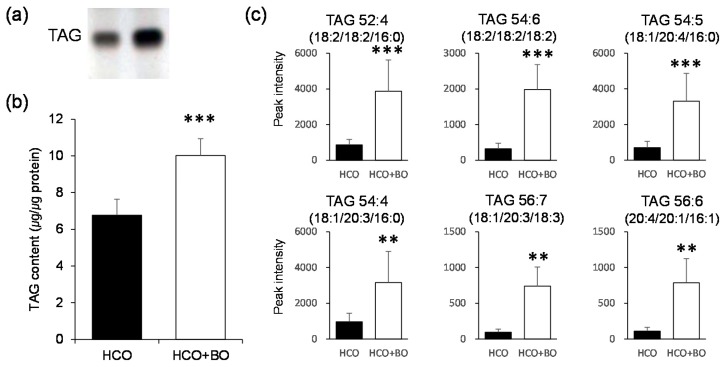
Triacylglycerol (TAG) content in the epidermis of guinea pigs fed a hydrogenated coconut oil (HCO) diet for 10 weeks (group HCO) or 8 weeks followed by 2 weeks of borage oil (BO) diet (group HCO + BO). Total TAG content was analyzed by high-performance thin-layer chromatography (HPTLC). (**a**) Representative band and (**b**) densitometric analysis. (**c**) Analysis of TAG species was performed by direct-infusion electrospray mass spectrometry (ES-MS). Values are means ± SD (*n* = 5). ** *p* < 0.01, *** *p* < 0.001 vs. group HCO by the unpaired Student’s *t* test.

First, we assessed the total TAG content in the epidermis of groups HCO and HCO + BO using HPTLC analysis (Figure 1a). The total TAG content was significantly increased in group HCO + BO compared with group HCO (Figure 1b), as reported previously [5]. To identify which TAG species were altered between groups HCO and HCO + BO, we used a lipidomic analysis approach (Figure 1c and Table S1). TAG 52:4 and TAG 54:6 containing LNA, TAG 54:4 and TAG 56:7 containing GLA and dihomo-γ-linolenic acid (DGLA; 20:3*n*-6), and TAG 54:5 and TAG 56:6 containing arachidonic acid (ARA; 20:4*n*-6) were significantly increased in group HCO + BO compared with group HCO. Other TAG species were not altered in either group (data not shown). These results indicate that the increased total TAG content in group HCO + BO may have resulted from an increase in TAG species containing EFA (i.e., LNA and GLA and their C20 metabolized fatty acids (FA) such as DGLA and ARA).

### 3.2. Altered Expression of Genes Related to TAG Synthesis

**Figure 2:**
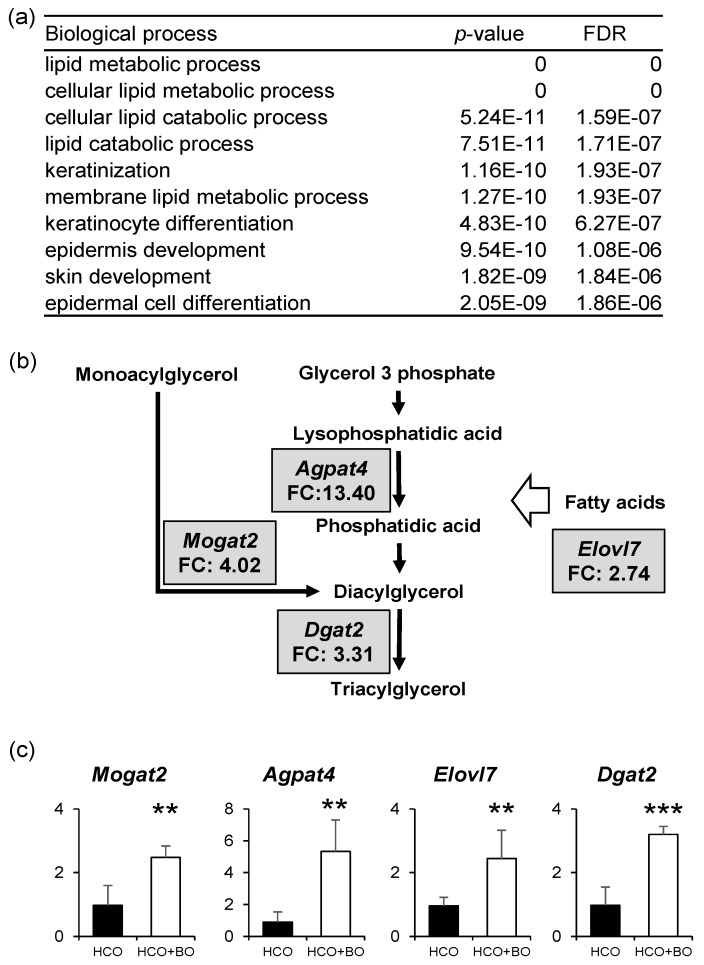
Gene expression levels in the epidermis of guinea pigs fed a hydrogenated coconut oil (HCO) diet for 0 weeks (group HCO) or 8 weeks followed by 2 weeks of borage oil (BO) diet (group HCO + BO). Transcriptomes were obtained by microarray analysis (*n* = 3). (**a**) The gene ontology enrichment analysis of up-regulated genes in group HCO + BO (Fold change; FC > 1.5) was performed using a web-based gene set analysis toolkit. Results are listed by false discovery rate (FDR) and the adjusted *p*-value is corrected for multiple comparisons. (**b**) Among genes related to triacylglycerol synthesis, up-regulated genes in group HCO + BO were selected (FC > 2). (**c**) The gene expression levels of monoacylglycerol O-acyltransferase 2 (*Mogat2*), 1-acylglycerol-3-phosphate O-acyltransferase 4 (*Agpat4*), and the elongation of very long-chain fatty acids 7 (*Elovl7*) and diacylglycerol O-acyltransferase 2 (*Dgat2*) were validated by a quantitative reverse transcription polymerase chain reaction (qRT-PCR, *n* = 5). Values are FC or means ± SD. ** *p* < 0.01, *** *p* < 0.001 vs. group HCO by the unpaired Student’s *t* test.

To identify the genes related to altered TAG content, we compared epidermal transcriptomes between groups HCO and HCO+BO using microarray analysis. First, we evaluated the overall meaning of up-regulated genes (FC > 1.5, *n* = 1243) in group HCO + BO (Table S2) by GO enrichment analysis (Figure 2a and Table S3). The up-regulated genes in group HCO + BO were involved in biological processes involved in the lipid metabolic process, keratinocyte differentiation, and epidermis development. Among the up-regulated genes associated with the lipid metabolic process (Table S4), we further checked genes related to TAG metabolism, especially with the two major pathways of TAG synthesis, the glycerophosphate pathway and monoacylglycerol (MAG) pathway [12]. As shown in Figure 2b, gene expression levels of monoacylglycerol O-acyltransferase 2 (*Mogat2*), 1-acylglycerol-3-phosphate O-acyltransferase 4 (*Agpat4*) and the elongation of very long-chain fatty acids 7 (*Elovl7*) and Dgat2 increased over two to thirteen-fold in group HCO + BO compared with group HCO; these results were confirmed by qRT-PCR (Figure 2c). Although there is little information about the MAG pathway in the epidermis, our results suggest that the MAG pathway seems to play a role in TAG synthesis in the epidermis, at least in guinea pigs. In the glycerophosphate pathway, *Agpat4* seems to play a control step in TAG synthesis in the guinea pig epidermis. Because *Agpat4*, an acyltransferase that transfers an acyl-CoA onto the sn-2 position of glycerol [1], has been reported to have a preference for a long-chain polyunsaturated fatty acyl-CoA (including 18:2 and 20:4-CoA) [13], the increase of the TAG species esterified LNA and ARA at the sn-2 position in group HCO + BO seems to be largely attributed to *Agpat4*. In addition, the increase in the TAG species esterified DGLA (the elongated C20 metabolized FA of GLA) in group HCO + BO might be attributed to the increased expression of *Elovl7*, which is known to have the highest activity toward 18:3-CoA [14]. *Dgat2*, which is responsible for the last step of TAG synthesis, is known to play a crucial role in skin barrier homeostasis [15]. Overexpression of *Dgat2* causes acyl-Cer synthesis [16], while *Dgat2-*deficient mice show decreased content of acyl-Cer [4], suggesting that the decreased CLE content in EFA-deficient epidermis [17] could be related to decreased *Dgat2* expression.

### 3.3. Altered Expression of Genes Related to TAG Hydrolysis and CLE Formation

To understand the implication of altered TAG content and gene expression related to TAG metabolism such as *Dgat2*, we further evaluated the genes involved in TAG hydrolysis, acyl-Cer synthesis, and CLE formation (Table 2). Among the various candidate genes that could hydrolyze TAG [15], the expression levels of lipase family member N (*Lipn*, FC = 3.66) and K (*Lipk*, FC = 3.65) and the patatin-like phospholipase domain containing 5 (*Pnpla5*, FC = 2.35) were increased in group HCO + BO. In addition, the expression levels of genes that induce the hydroxylation of ultra-long-chain FA (*Cyp4f22*), acylation of FA into sphingoid bases (*Cers3*), transportation of lamellar granules (*Abca12*), and oxidation of LNA for CLE formation (*Alox12b* and *Aloxe3*) [18] were also increased approximately 1.5–3-fold (Table 2). Collectively, these results suggest that the EFA esterified into TAG is likely to be hydrolyzed by these lipases and esterified for acyl-Cer synthesis, which is subsequently utilized for CLE formation by these genes. Furthermore, because *Alox12b*, the dioxygenase required for the formation of covalent bond between acyl-Cer and corneocytes, has been reported to have a preference for GLA as well as LNA [19], it could be speculated that GLA-esterified acyl-Cer, which was reported in our previous studies, might be involved in maintaining the integrity of CLE. Therefore, the genes listed in Table 2 seem to be related to restoring the impaired skin barrier in EFA-deficient guinea pigs by BO supplementation [6].

## 4. Conclusions

Dietary BO induced the increase of TAG content and TAG species esterified LNA, GLA, DGLA, and ARA in parallel with the up-regulation of the expression of genes involved in TAG synthesis including *Mogat2*, *Agpat4*, *Elovl7*, and *Dgat2* as well as TAG hydrolysis, acyl-Cer synthesis, and CLE formation in the epidermis of EFA-deficient guinea pigs. These results suggest that TAG could serve as an important EFA-donor required for acyl-Cer synthesis.

## Figures and Tables

**Table 1:** qRT-PCR primer sequences.

Gene ^1^	Forward	Reverse
*Gapdh*	5′-AGAACATCATCCCCGCATCC-3′	5′-TCCACAACCGACACATTAGGT-3′
*Mogat2*	5′-TGCTCTACCTTTTGCTTATGGG-3′	5′-TGGCTTGTCTCGGTCCA-3′
*Agpat4*	5′-GCTGATTGTTATGTTAGGCGGA-3′	5′-GACTTTGGGGGTTTCTGGGA-3′
*Elovl7*	5′-GGACAGAGTTCCAGCGAGTA-3′	5′-ACAAGTGAGAGTCAAAAGCCTG-3′
*Dgat2*	5′-CTCCTCTGTCAAATCTCAGGC-3′	5′-TTACTCCAACAACACGCAGG-3′

^1^*Gapdh*: glyceraldehyde 3-phosphate dehydrogenase (NCBI Accession: NM_001172951), *Mogat2*: monoacylglycerol O-acyltransferase 2 (NCBI Accession: XM_003468553), *Agpat4*: 1-acylglycerol-3-phosphate O-acyltransferase 4 (NCBI Accession: XM_003466380), *Elovl7*: elongation of very long-chain fatty acids 7 (NCBI Accession: XM_003470212) and *Dgat2*: diacylglycerol O-acyltransferase 2 (NCBI Accession: XM_003468552).

**Table 2:** Up-regulated genes associated with epidermal lipase, acyl-Cer synthesis and CLE formation in group HCO + BO ^1.^

Gene ^2^	FC ^3^	Function
*Lipn*	3.66	Lipases
*Lipk*	3.65	Lipases
*Pnpla5*	2.35	Lipases
*Elovl4*	1.72	Elongation of fatty acids
*Cyp4f22*	1.57	Omega-hydroxylation of ultra-long-chain fatty acids
*Cers3*	1.61	Ceramide synthesis
*Ugcg*	1.53	Glucosylation of ceramide
*Abca12*	3.07	Transport via lamellar granules
*Alox12b*	2.13	Oxidation of linoleic acid in ceramide
*Aloxe3*	1.65	Oxidation of linoleic acid in ceramide

^1^ Cer: ceramide, CLE: corneocyte lipid envelope, group HCO+BO: hydrogenated coconut oil (HCO) diet for 8 weeks followed by borage oil (BO) diet for 2 weeks. ^2^ *Lipn*: lipase family member N, *Lipk*: lipase family member K, *Pnpla5*: patatin-like phospholipase domain containing 5, *Elovl4*: elongation of very long-chain fatty acids 4, *Cyp4f22*: cytochrome P450 family 4 subfamily F member 22, *Cers3*: ceramide synthase 3, *Ugcg*: UDP-glucose ceramide glucosyltransferase, *Abca12*: ATP binding cassette subfamily A, *Alox12b*: arachidonate 12-lipoxygenase 12R type, *Aloxe3*: arachidonate lipoxygenase 3. ^3^ 3-fold change (FC) of group HCO + BO with respect to group HCO (HCO diet for 10 weeks).

## Supplementary Materials

The following are available online at https://www.mdpi.com/2072-6643/11/11/2818/s1, Table S1: Experimental *m*/*z* and signal intensities of each triacylglycerol species quantitated, Table S2: Up-regulated gene list in HCO + BO (fold change; FC > 1.5), Table S3: Over-representation analysis with up-regulated genes in HCO + BO, Table S4: Up-regulated genes in HCO + BO related to GO: 0006629: lipid metabolic process.

## References

[1] Lu B., Jiang Y.J., Man M.Q., Brown B., Elias P.M., Feingold K.R. (2005). Expression and regulation of 1-acyl- sn -glycerol- 3-phosphate acyltransferases in the epidermis. J. Lipid Res..

[2] Radner F.P.W., Streith I.E., Schoiswohl G., Schweiger M., Kumari M., Eichmann T.O., Rechberger G., Koefeler H.C., Eder S., Schauer S. (2010). Growth Retardation, Impaired Triacylglycerol Catabolism, Hepatic Steatosis, and Lethal Skin Barrier Defect in Mice Lacking Comparative Gene Identification-58 (CGI-58). J. Biol. Chem..

[3] Ujihara M., Nakajima K., Yamamoto M., Teraishi M., Uchida Y., Akiyama M., Shimizu H., Sano S. (2010). Epidermal triglyceride levels are correlated with severity of ichthyosis in Dorfman–Chanarin syndrome. J. Dermatol. Sci..

[4] Stone S.J., Myers H.M., Watkins S.M., Brown B.E., Feingold K.R., Elias P.M., Farese R. (2004). V Lipopenia and skin barrier abnormalities in DGAT2-deficient mice. J. Biol. Chem..

[5] Bibel D.J., Miller S.J., Brown B.E., Pandey B.B., Elias P.M., Shinefield H.R., Aly R. (1989). Antimicrobial activity of stratum corneum lipids from normal and essential fatty acid-deficient mice. J. Invest. Dermatol..

[6] Chung S., Kong S., Seong K., Cho Y. (2002). γ-Linolenic Acid in Borage Oil Reverses Epidermal Hyperproliferation in Guinea Pigs. J. Nutr..

[7] Shin K.-O., Kim K., Jeon S., Seo C.-H., Lee Y.-M., Cho Y. (2015). Mass Spectrometric Confirmation of γ-Linolenic Acid Ester-Linked Ceramide 1 in the Epidermis of Borage Oil Fed Guinea Pigs. Lipids.

[8] Kim K.-P., Jeon S., Kim M.-J., Cho Y. (2018). Borage oil restores acidic skin pH by up-regulating the activity or expression of filaggrin and enzymes involved in epidermal lactate, free fatty acid, and acidic free amino acid metabolism in essential fatty acid-deficient Guinea pigs. Nutr. Res..

[9] Shin J., Kim Y.J., Kwon O., Kim N.-I., Cho Y. (2016). Associations among plasma vitamin C, epidermal ceramide and clinical severity of atopic dermatitis. Nutr. Res. Pract..

[10] Shon J.C., Shin H.-S., Seo Y.K., Yoon Y.-R., Shin H., Liu K.-H. (2015). Direct infusion MS-based lipid profiling reveals the pharmacological effects of compound K-reinforced ginsenosides in high-fat diet induced obese mice. J. Agric. Food Chem..

[11] Liao Y., Wang J., Jaehnig E.J., Shi Z., Zhang B. (2019). WebGestalt 2019: gene set analysis toolkit with revamped UIs and APIs. Nucleic Acids Res..

[12] Jiang Y.J., Feingold K.R. (2011). The expression and regulation of enzymes mediating the biosynthesis of triglycerides and phospholipids in keratinocytes/epidermis. Dermatoendocrinol..

[13] Eto M., Shindou H., Shimizu T. (2014). A novel lysophosphatidic acid acyltransferase enzyme (LPAAT4) with a possible role for incorporating docosahexaenoic acid into brain glycerophospholipids. Biochem. Biophys. Res. Commun..

[14] Naganuma T., Sato Y., Sassa T., Ohno Y., Kihara A. (2011). Biochemical characterization of the very long-chain fatty acid elongase ELOVL7. FEBS Lett..

[15] Radner F.P.W., Fischer J. (2014). The important role of epidermal triacylglycerol metabolism for maintenance of the skin permeability barrier function. Biochim. Biophys. Acta - Mol. Cell Biol. Lipids.

[16] Ohno Y., Kamiyama N., Nakamichi S., Kihara A. (2017). PNPLA1 is a transacylase essential for the generation of the skin barrier lipid ω-O-acylceramide. Nat. Commun..

[17] Elias P.M., Gruber R., Crumrine D., Menon G., Williams M.L., Wakefield J.S., Holleran W.M., Uchida Y. (2014). Formation and functions of the corneocyte lipid envelope (CLE). Biochim. Biophys. Acta - Mol. Cell Biol. Lipids.

[18] Akiyama M. (2017). Corneocyte lipid envelope (CLE), the key structure for skin barrier function and ichthyosis pathogenesis. J. Dermatol. Sci..

[19] Sun D., McDonnell M., Chen X.-S., Lakkis M.M., Li H., Isaacs S.N., Elsea S.H., Patel P.I., Funk C.D. (1998). Human 12(R)-Lipoxygenase and the Mouse Ortholog. J. Biol. Chem..

